# Supplementary material for: Splice-Junction-Based Mapping of Alternative Isoforms in the Human Proteome
Source: Cell Rep. Author manuscript; Available in PMC 2020 Jan 15. (PMC6961840; doi:10.1016/j.celrep.2019.11.026)
Supplement: 3 [file NIHMS1546469-supplement-3.zip › DF2/PXD0010154/Ovary-75-Q12805-RGEQCVDIDECR.pdf]

A

Predicted sequence disorder and sequence features of Q12805

Peptide: RGEQCVDIDECR Junction: sp|Q12805|FBLN3_HUMAN|ENSG00000115380|SE2|32779|chr2|55875065|55877865|−2|r32|T1 TrNovel: FALSE

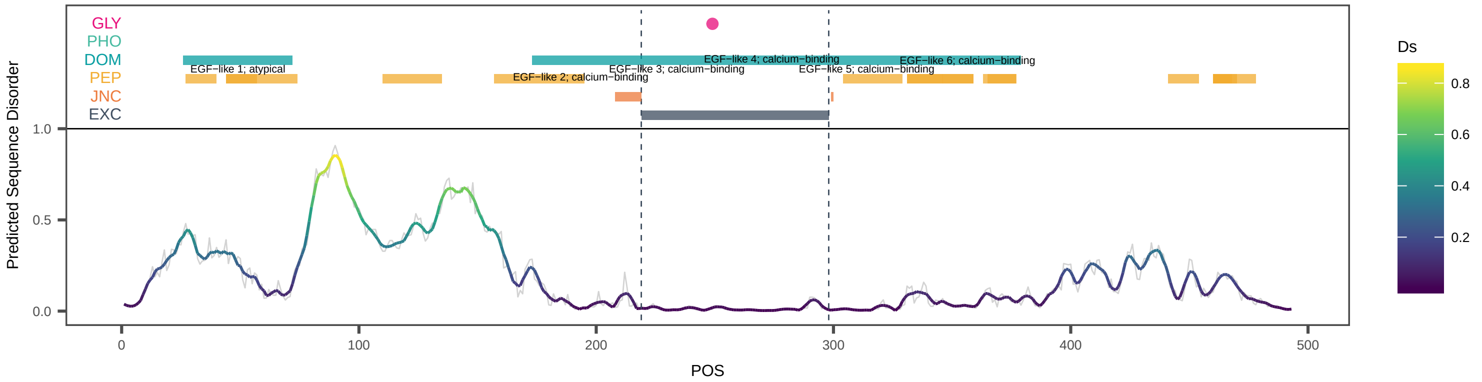

B

Distribution of sequence disorder in excised vs. mapped and non−excised regions of protein

M−W P−value vs. mapped: 6.81e−29 vs. non−excised: 2.35e−35

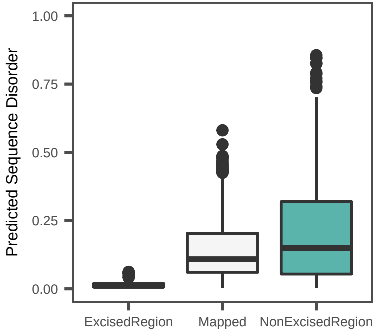

C
